# New method of peptide cleavage based on Edman degradation

**DOI:** 10.1007/s11030-013-9453-y

**Published:** 2013-05-21

**Authors:** Remigiusz Bąchor, Alicja Kluczyk, Piotr Stefanowicz, Zbigniew Szewczuk

**Affiliations:** Faculty of Chemistry, University of Wrocław, ul. F. Joliot-Curie 14, 50-383 Wrocław, Poland

**Keywords:** OBOC, Ionization tag, Quaternary ammonium salts, Peptide cleavage method, Phenylthiohydantoin, Edman degradation

## Abstract

A straightforward cleavage method for *N*- acylated peptides based on the phenylthiohydantoin (PTH) formation is presented. The procedure could be applied to acid-stable resins, such as TentaGel HL–NH$_{2}$. We designed a cleavable linker that consists of a lysine residue with the $\upalpha$-amino group blocked by Boc, whereas the $\upvarepsilon$-amino group is used for peptide synthesis. After the peptide assembly is completed, the protecting groups in peptide side chains are removed using trifluoroacetic acid, thus liberating also the $\upalpha$-amino group of the lysine in the linker. Then the reaction with phenyl isothiocyanate followed by acidolysis causes an efficient peptide release from the resin as a stable PTH derivative. Furthermore, the application of a fixed charge tag in the form of 2-(4-aza-1-azoniabicyclo[2.2.2]octylammonium)acetyl group increases ionization efficiency and reduces the detection limit, allowing ESI-MS/MS sequencing of peptides in the subfemtomolar range. The proposed strategy is compatible with standard conditions during one-bead-one-compound peptide library synthesis. The applicability of the developed strategy in combinatorial chemistry was confirmed using a small training library of $\upalpha$-chymotrypsin substrates.

**Electronic supplementary material:**

The online version of this article (doi:10.1007/s11030-013-9453-y) contains supplementary material, which is available to authorized users.

## Introduction

Combinatorial chemistry is widely used in the fast search for new biologically active compounds [1, 2]. Particularly useful are combinatorial one-bead-one-compound (OBOC) peptide libraries, obtained by the split-and-mix technique that allows rapid synthesis of millions of compounds on solid support and screening for their biological activity. The screening of OBOC combinatorial peptide libraries is usually carried out with the deprotected peptide remaining attached to the resin bead. After a hit is identified, the respective bead is isolated and the peptide can be sequenced on-resin by Edman degradation or can be cleaved from the bead for sequencing by mass spectrometry.

Routinely, the screening is performed on peptides attached to PEGyleted resin such as TentaGel. This hybrid resin is not sensitive to acids or bases [3, 4]; therefore, after deprotection of side-chain groups with trifluoroacetic acid (TFA), the peptide is still attached to the solid support. For that reason, a special cleavage method is necessary to release the peptides from selected single beads. This requires application of a cleavable linker attached to the C-terminal part of the investigated peptide. Essentially, any photocleavable or chemically cleavable functional group can be used as the linker, as long as it is fully compatible with the reagents used in library synthesis and screening. A methionine residue is often used as a linker because its cleavage by cyanogen bromide (CNBr) is rather specific and efficient, whereas the resulting homoserine lactone is chemically stable [5, 6]. However, the disadvantages of this cleavage method are high toxicity of CNBr as well as the necessity of excluding Met and Trp from the diversity pool of the library. In addition, spontaneous oxidation of methionine, that may occur upon storage and handling, leads to methionine sulfoxide, which is not cleavable by CNBr. Another linker containing the Asp-Pro sequence has been proposed by Masforrol et al. [7]. Although this linker is effectively cleavable by acids, it also eliminates the Asp-Pro sequence and other unnatural acid-sensitive amino acids from the diversity set. Other cleavable linkers, including a modified glycolic acid anchor [8] or photolabile nitrophenyl derivatives [9], are rather expensive, their cleavage methods may require tedious desalting of released peptides before MS sequencing or they are not fully compatible with some photolabile amino acids. Therefore, a search for a new efficient method of peptide cleavage from a single bead of TentaGel HL–NH$_{2}$ resin is required.

Regular Edman sequencing [10] can be used for the on-bead sequence analysis without cleaving off and retrieving the peptide [11, 12]. However, this procedure is time-consuming, expensive, restricted to $\upalpha$-amino acid residues only, and requires a free N-terminal amino group. This makes the method not suitable for analysis of libraries containing some modified amino acids and other organic building blocks commonly used in combinatorial chemistry.

For OBOC peptide libraries released from support, matrix-assisted laser desorption/ionization (MALDI-MS) as well as electrospray (ESI-MS) mass spectrometry have been successfully used for sequencing by the combination of “ladder-synthesis” [13] or “ladder-sequencing” [14]. However, these methods present several disadvantages such as a long time of complicated library preparation and misinterpretation of some byproducts resulting from the peptide modification. The fundamental problem of OBOC peptide library analysis is the small amount of compound obtained from a single resin bead, and insufficient ionization efficiency of some peptides for standard ESI–MS analysis. Therefore, application of peptide ionization enhancers in a form of fixed charge tags may enable the analysis of peptide obtained from a single resin bead. Recently, we developed an efficient and straightforward method for quaternary ammonium salt (QAS) formation on solid support [15]. Such modification facilitates MS analysis of peptides, allowing for ESI-MS/MS sequencing of trace amounts of compounds by both charge remote and charge directed fragmentation mechanisms [16], even in case of peptides lacking easily mobilizable protons [17]. Our previous work presented the application of QAS for high-throughput analysis of single resin beads from OBOC peptide libraries using high-resolution electrospray ionization tandem mass spectrometry (HR ESI-MS/MS) [18]. The proposed cleavable linker was composed of lysine with the $\upvarepsilon$-amino group labeled by QAS and methionine, which allows selective cleavage of the peptide from TentaGel resin by CNBr. To overcome the problems associated with that procedure, we propose a new strategy of peptide cleavage from TentaGel HL–NH$_{2}$ resin based on the Edman degradation method.

Edman degradation [10] is commonly used for peptide sequencing both in solution and on solid support. The method is compatible with natural as well as non-natural $\upalpha$-amino acid residues located at the N-terminus, as long as they have the $\upalpha$-amino group unprotected [19]. The reaction with phenyl isothiocyanate (PITC) under mildly alkaline conditions, followed by acidolysis, results in the cleavage of the N-terminal residue as phenylthiohydantoin (PTH) derivative without disrupting the peptide bonds between other amino acid residues. This method does not affect functional groups in amino acid side chains (with exception of the $\upvarepsilon$-amino group of lysine, which forms a stable phenylthiourea (PTU) derivative) [20]. The usefulness of this method is based on high yield of formation of amino acid-PTH, suitable for identification by using chromatography, electrophoresis, or mass spectrometry.

Our approach is based on application of the $\upvarepsilon$-amino group of lysine for peptide assembly, whereas the $\upalpha$ amino group after reaction with PITC allows cyclative cleavage by PTH formation (e.g., Edman degradation). Although the mechanism of Edman degradation is well known and this procedure is generally accepted for analysis of peptides attached to solid support [12], to the best of our knowledge, the method has not been applied for cleavage of whole peptide-PTH derivatives from a resin.

## Results and discussion

**Fig. 1:**
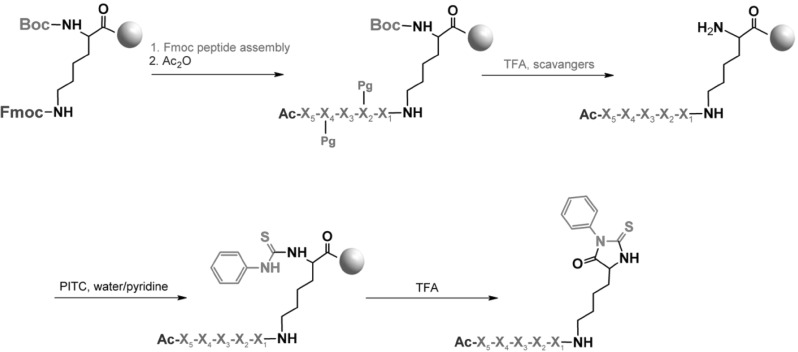
Proposed peptide cleavage method based on Edman degradation. *BOC* *tert*-butyloxycarbonyl, *Fmoc* 9-fluorenylmethoxycarbonyl, *Pg* protecting group, $X_1$–$X_5$ amino acid residues, *Ac* acetyl group, *PITC* phenyl isothiocyanate. A *gray ball* represents a single resin bead

We developed a straightforward method of cleavage of N-acylated peptides from TentaGel HL–NH$_{2}$ resin based on the well-known PTH formation reaction commonly used in peptide sequencing by the Edman degradation method. The method utilizes an acid cleavable linker consisting of a lysine residue, where the $\upalpha$-amino group is blocked with a Boc group whereas the $\upvarepsilon$-amino group is used for peptide synthesis according to Fmoc strategy [21]. The deprotection of side chains with TFA at the same time liberates the $\upalpha$-amino group of the lysine residue which in turn reacts with PITC. The final form of the linker consists of a lysine residue with the $\upalpha$-amino group blocked by a phenylthiocarbonyl group and an N-acylated peptide assembled on the $\upvarepsilon$-amino group (Fig. 1). The subsequent acidolysis leading to Edman degradation results in formation of a stable PTH derivative and efficient peptide release from the resin, allowing identification of the peptide sequence by mass spectrometry, which is especially useful in the analysis of hits in OBOC peptide libraries.

To validate the proposed approach, the TentaGel HL–NH$_{2}$ resin was coupled with Boc-Lys(Fmoc)–OH. After removal of the Fmoc group, the peptide chain was assembled on the $\upvarepsilon$ amino group according to standard Fmoc strategy and the N-terminal residue was acetylated. TFA treatment of the resulting peptidyl resin deprotected all side-chain functional groups including the Boc protecting group from the $\upalpha$-amino group of the lysine linker. The resulting peptidyl resin was treated with PITC in water/pyridine mixture (1:1 v/v), according to the method described by Edman [10]. During the optimization process, we found that microwave-assisted formation of PTU peptide derivative is significantly faster, reducing reaction time to 1 min at 70 $^\circ$C as compared to classical methods [22]. Carrying the PTU synthesis on solid support allowed application of large excess (20 fold) of PITC. We confirmed the completeness of PITC attachment to the $\upalpha$-amino group of the lysine linker by ninhydrin test. The resulted resin containing the PTU peptide derivative can be used for combinatorial screening. The application of TFA (30 min at room temperature) leads to closing of PTH ring which causes the removal of the peptide-PTH derivative from the resin (Fig. 1). The efficiency of the proposed cleavage method was confirmed by elemental analysis of peptidyl TentaGel resin. The comparison of sulfur content in resin samples measured before (2.83 %) and after incubation with TFA (0 %) suggests high efficiency of our cleavage method based on Edman degradation.

To confirm the cleavage efficiency of the proposed methodology, a new bifunctional linker, allowing peptide cleavage in two different ways was designed (supporting materials, Fig. S1). This linker consists of Lys-Phe-Met fragment: in the lysine residue, the $\upvarepsilon$-amino group is used for further peptide assembly, whereas the $\upalpha$-amino functionality provides a phenyl isothiocyanate attachment point and allows peptide cleavage as PTH. The phenylalanine residue is introduced to increase the UV detection sensitivity and the methionine residue allows peptide cleavage using CNBr. The model peptide attached to such linker was modified by PITC, washed and dried in vacuo, as described in the Experimental section. A resin sample (20 mg) containing PTU-peptide derivative was treated with TFA for 30 min, the solution was evaporated and lyophilized. The remaining resin was treated with CNBr (as described in our previous paper [18]). If the peptide is completely cleaved by PTH formation, in the mass spectrum of the product we should observe only signals corresponding to the Phe-HSL (HSL—homoserine lactone). As a control sample we used the resin modified by PITC, which was treated with 0.25M CNBr in 70 % HCOOH overnight. MS and HPLC analysis of the product revealed only the presence of peptidic PTH and Phe-HSL. The obtained chromatograms (supporting materials, Figs. S2–S3) demonstrated complete peptide cleavage efficiency of the new proposed method after 30-min incubation with TFA.

To test the applicability of the proposed peptide cleavage method in combinatorial chemistry, a small training library of $\upalpha$-chymotrypsin substrates was synthesized on TentaGel HL–NH$_{2}$. According to our previous studies concerning on-resin peptide library design and analysis strategy [18], we included into the linker a QAS moiety as an ionization tag; however, instead of a previously used standard method, based on methionine as a linker cleavable by CNBr, we used the proposed cleavage method based on the Edman degradation.

**Fig. 2:**
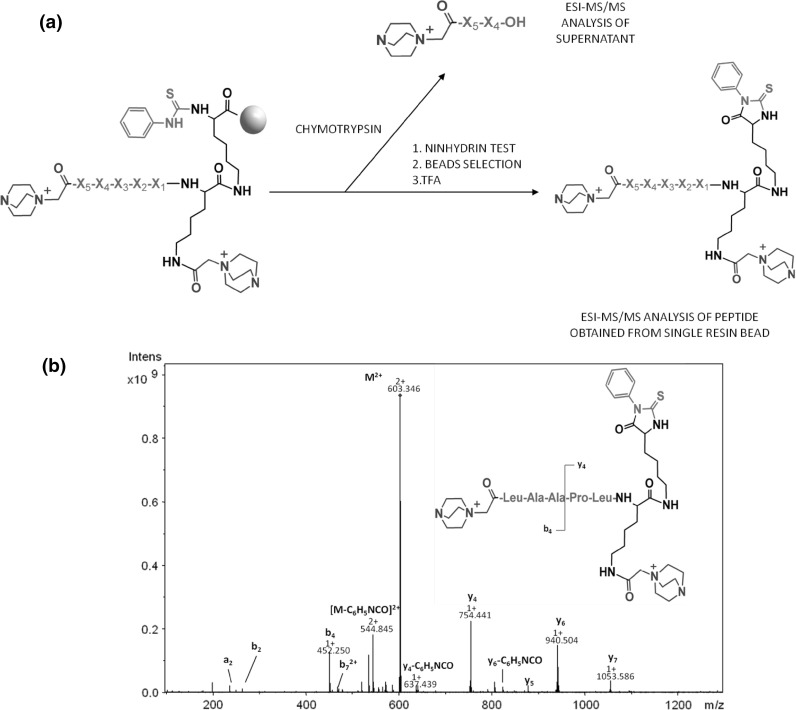
Application of a new strategy for OBOC combinatorial library synthesis and analysis: **a** the fixed charge tag located at the N-terminus allows sensitive supernatant analysis. A *gray ball* represents a single resin bead. **b** the ESI-MS/MS spectrum of library component released from a single resin bead using the new peptide cleavage method. Parent ion was 603.346 $\text{ M }^{2+}$

The resin was modified by the linker composed of Boc-Lys(Fmoc-Lys(Mtt)), allowing introduction of the fixed charge tag for sensitive ESI-MS/MS analysis, in the form of 2-(4-aza-1-azoniabicyclo-[2.2.2]octylammonium)acetyl group (Aabo-acetyl) on the $\upvarepsilon$-amino group of lysine residue after Mtt removal (Fig. 2). A second Aabo-acetyl group was attached to the N-terminal residues after the synthesis of the peptide library. This modification protects the N-terminal amino groups from ninhydrin reaction in peptides resistant to chymotrypsin, and provides a fixed charge tag allowing sensitive detection and identification of fragments in the supernatant obtained after enzymatic digestion performed even on single resin bead (Spectrum S2).

The presence of two fixed charge ionization tags allows sensitive analysis by mass spectrometry of both peptides present in supernatant after incubation with $\upalpha$-chymotrypsin as well as remaining on solid support, as we described in our previous work [18]. A combinatorial OBOC library was synthesized according to Fmoc strategy, using split-and-mix technique [18]. A pentapeptide fragment with the Leu-Tyr-Gln-Leu-Glu sequence, corresponding to the insulin A chain (13–17), which was identified as a chymotrypsin substrate [23], responsible for amyloids plaque formation [24], was selected as a model peptide. Because of the amino acid residue selection criteria [18] used in library design, most of the library components turned out to be $\upalpha$-chymotrypsin substrates. After deprotection and reaction with PITC, the obtained library of resin-bound PTU modified peptides was subjected to enzymatic digestion by $\upalpha$-chymotrypsin. In a separate experiment we confirmed that PTU derivatives are stable at pH 7.8, which allows application of MS compatible ammonium bicarbonate buffer. After supernatant separation and thorough bead washing, the simple and efficient ninhydrin test was used for hit identification by detection of primary amino groups obtained after enzymatic digestion [25].

The main goal of the designed library was to verify the suitability of the proposed new peptide cleavage method in combinatorial chemistry by determination of the peptide sequences obtained from both the positive and negative hits. 50 purple resin beads (positive hits) and 50 yellow resin beads (negative hits) were selected for HR ESI-MS/MS analysis. Each bead was treated independently with TFA for 30 min, releasing PTH peptide derivatives. The procedure, including enzymatic digestion and peptide cleavage, is presented in Fig. 2.

All selected beads were decoded producing multiple confirmations for the sequences of peptides. The obtained mass spectra (Supplementary data) show signals corresponding to the undigested peptides, which may suggest that only a small amount of the available peptides was digested on-resin; however, it is sufficient to produce a positive response in the ninhydrin test. The incomplete proteolysis is actually an advantage, since the amount of the intact peptide on the bead available for MS/MS analysis after chemical cleavage is not significantly reduced. The application of ninhydrin test for positive hit identification limits library diversity as lysine and arginine cannot be included in library sequences due to color reaction with ninhydrin. However, application of other identification method would not exclude the basic amino acids from the library pool, and would not require blocking of the linker $\upalpha$-amino group by PTU.

The ESI-MS/MS spectrum (Fig. 2b) shows a series of *a*-, *b*-, and *y*-type ions which unambiguously identify the sequence of peptide obtained from a single resin bead by our cleavage procedure. The introduction of two QAS Aabo-acetyl motives results in interpretable mass spectra allowing peptide identification at low femtomolar level. A fragmentation within the PTH-amino acids, which has been described previously [26] was also observed. Additionally, characteristic elimination of $\text{ C }_{6}\text{ H }_{5}$NCO ion can simplify the MS/MS spectrum interpretation. No peaks corresponding to the QAS group fragmentation or elimination were observed. Fragmentation spectra recorded for other library members are presented in supplementary data.

The proposed cleavage procedure was developed for analytical application in combinatorial chemistry. However, some peptide-thiohydantoin conjugates were reported to block TRPV1 ion channels [27], and our method could be applied for preparation of C-terminally modified peptides for biological studies.

## Conclusions

A straightforward method for cleavage of peptides from TentaGel HL–NH$_{2}$ resin, based on Edman degradation has been proposed. The N-acylated peptide is assembled via Fmoc strategy on Boc-Lys(Fmoc) coupled to the resin. Deprotection of the peptide side chains as well as the $\upalpha$-amino group of the lysine residue in the linker with TFA followed by reaction with PITC and subsequent acidolysis allows cleavage of the PTH-peptide-acetate from the resin. The obtained PTH-peptide derivatives containing the ionization tag can be easily sequenced by ESI-MS/MS method. The optimized microwave-assisted procedure significantly reduces the time of PTU derivative formation. The proposed method is compatible with standard solid-phase peptide synthesis, does not require special equipment nor handling of very toxic reagents. The application of commercially available Boc-Lys(Fmoc)-OH as a building block for the cleavage linker, and the widely used Edman reagent significantly reduce the costs of library synthesis, as compared to the use of expensive photolabile linkers or the methionine/CNBr method. The introduction of the fixed charge tag at N-terminal residues of library components allowed unambiguous identification of N-terminal proteolysis products in supernatant, although concentration of such products is very low.

The presented methodology may be particularly useful in sequencing peptides attached to a single resin bead in OBOC combinatorial peptide libraries.

## Experimental procedures

All solvents and reagents were used as supplied. Fmoc amino acid derivatives (Fmoc-Leu–OH, Fmoc-Tyr(Bu$^\mathrm{t}$)–OH, Fmoc-Gln(Trt)–OH, Fmoc-Glu(OBu$^\mathrm{t}$)–OH, Fmoc-Asp (OBu$^\mathrm{t}$)–OH, Fmoc-Pro–OH, Fmoc-His(Trt)–OH, Fmoc-Lys (Mtt)–OH, Fmoc-Val–OH, Fmoc-Thr(Bu$^\mathrm{t}$)–OH) and Boc-Lys(Fmoc)–OH were purchased from Novabiochem or IrisBiotech; TentaGel®HL–NH$_{2}$ resin (0.56 mmol/g, 110 $\upmu$m particle size) was purchased from Rapp Polymere. Benzotriazole-1-yl-oxy-tris-pyrrolidinophosphonium hexafluorophosphate (PyBOP) was obtained from Novabiochem, TFA was from IrisBiotech. 1,4-Diazabicyclo[2.2.2]octane (DABCO), diisopropylethylamine (DIEA), PITC, and solvents for peptide synthesis: dimethylformamide (DMF) and dichloromethane (DCM) were obtained from Aldrich; alpha-chymotrypsin in crystal form (activity $\ge$40 units BTEE/mg of protein, with the addition of trypsin inhibitor TLCK) from bovine pancreas, Aldrich; pyridine, methanol (MeOH) and acetonitrile (MeCN) were purchased from POCH; iodoacetic acid from Merck; *N*,*N*-diisopropylcarbodiimide (DIC), triisopropylsilane (TIS), and ammonium bicarbonate were purchased from Fluka.

### Synthesis

TentaGel HL–NH$_{2}$ resin (50 mg, 28 $\upmu$mol) was suspended in DMF (1 mL) for 30 min. A mixture of Boc-Lys(Fmoc)–OH derivative (39.4 mg, 84 $\upmu$mol), PyBOP (44 mg, 84 $\upmu$mol), and DIEA (31 $\upmu$L, 168 $\upmu$mol) was added and mixed for 2 h. Then the $\upvarepsilon$-amino group was used for peptide assembly according to Fmoc strategy. The $\upalpha$-amino groups of N-terminal amino acid residues were acetylated using ($\text{ Ac }_{2}$O/DMF/DIEA; 1:7.5:1.5). Side-chain protecting groups were cleaved with the mixture of TFA/TIS/H$_2$O (95:2.5:2.5; v:v:v) for 2 h and the peptidyl resin was washed with 5 % solution of DIEA/DMF (3 $\times$ 1 min), DMF (6 $\times$ 1 min), DMF/DCM (1:1; v:v, 1 min), DCM (6 $\times$ 1 min), DCM/MeOH (1:1; v:v, 1 min), and MeOH (6 $\times$ 1 min) and dried in vacuo.

#### QAS formation

QAS formation was performed according to the method described previously [28]. After peptide synthesis the $\upalpha$-amino groups of N-terminal amino acid residues were deprotected. Then, the Mtt protecting group was cleaved by using 1 % solution of TFA in DCM ($3 \times 2$ min, $3 \times 10$ min, $3 \times 2$ min) [18]. The peptidyl resin was washed with DCM ($3 \times 1$ min), DCM/DMF (1:1; v:v, 1 min), 5 % DIEA in DMF ($3 \times 1$ min), and DMF (7 $\times$ 1 min). The mixture of iodoacetic acid (52 mg, 280 $\upmu$mol) and DIC (35 mg, 280 $\upmu$mol), dissolved in DMF (0.5 mL), was added to the peptidyl resin (50 mg, 28 $\upmu$mol) and the reaction was allowed to proceed for 3 h. Then DABCO (63 mg, 560 $\upmu$mol), dissolved in DMF (0.5 mL), was added to the reaction vessel and mixed for 24 h.

#### Peptide cleavage

After side-chain protecting groups cleavage, 50 mg of TentaGel resin (28 $\upmu$mol) was swollen for 30 min in water and then washed with water ($3 \times 1$ min) and water/pyridine ($3 \times 1$ min, 1:1). Free amino groups ($\upalpha$-amino groups of lysine residues—see Fig. 1) were modified by phenyl isothiocyanate (146 $\upmu$L, 560 $\upmu$mol) in the mixture of water/pyridine (1:1; v:v) using CEM microwave reactor (T $=$ 70 $^\circ$C; *E* $=$ 30 W). The reaction proceeded for 1 min. The resin was washed with water/pyridine ($6 \times 1$ min), water/MeOH ($1 \times 1$ min), and MeOH ($4 \times 1$ min). The reaction was controlled by ninhydrin test. Then the resin was dried in vacuo. The PTU peptide derivatives bound to the resin were treated with TFA (1 mL, 30 min), which resulted in peptide cleavage through formation of PTH derivatives. Then TFA was removed in stream of nitrogen. The obtained PTH-peptide derivatives were dissolved in the mixture (70 $\upmu$L) of water, acetonitrile, and formic acid (50:50:0.1; v:v:v) and their structures were confirmed by HR-ESI-MS/MS (Spectrum S1, presented as an example).

#### Enzymatic digestion

The peptidyl resin (5 mg) containing PTU peptide derivatives (obtained in the reaction with PITC), was washed with water ($3 \times 1$ min) and 0.01 M ammonium bicarbonate in water ($3 \times 1$ min). Enzymatic digestion was performed in 0.01 M ammonium bicarbonate buffer for 1 h at 37 $^\circ$C. The peptidyl resin to enzyme ratio was 1,000:1. After the supernatant separation, the resin was washed with water ($3 \times 1$ min), acetonitrile ($3 \times 1$ min), and methanol ($3 \times 1$ min). Then two drops of each component (A: 80 g phenol in 20 mL ethanol, B: 2 mL 0.001 M KCN in 98 mL pyridine, and C: 5 g of ninhydrin in 100 mL ethanol) were added and incubated for 3 min at 100 $^\circ$C. The resin was washed with methanol ($3 \times 1$ min) and DMF ($3 \times 1$ min). Positive (purple beads) and negative (yellow beads) hits were separated manually, washed with water ($3 \times 1$ min), acetonitrile ($3 \times 1$ min), and methanol ($3 \times 1$ min) and dried in vacuo. Each selected bead was treated individually with TFA for 10 min. Then TFA was removed in stream of nitrogen. The obtained PTH-peptide derivatives were dissolved in the mixture (70 $\upmu$L) of water, acetonitrile, and formic acid (50:50:0.1; v:v:v) and analyzed by HR-ESI-MS/MS.

#### Mass spectrometry

All MS experiments were performed on an FT-ICR (Fourier Transform Ion Cyclotron Resonance) MS Apex-Qe Ultra 7T instrument (Bruker Daltonics, Germany) equipped with standard ESI source. Spectra were recorded for samples dissolved in the mixture of water, acetonitrile, and formic acid (50:50:0.1; v:v:v). The instrument was operated in the positive ion mode and calibrated before each analysis with the Tunemix$^\mathrm{TM}$ mixture (Bruker Daltonics, Germany) in quadratic method. The instrument parameters were as follows: scan range: 100–1,600 *m*/*z*; drying gas: nitrogen; flow rate: 1.5 L/min; dry gas (nitrogen) flow: 4 L/min; analyte solutions (70 $\upmu$L) were introduced at a flow rate of 3 $\upmu$L/min; temperature: 200 $^\circ$C; potential between the spray needle and the orifice: 4.2 kV. In the MS/MS experiments, the doubly charged $\text{ M }^{2+}$ precursor ions were selected on the quadrupole and subsequently fragmented in the hexapole collision cell. Argon was used as a collision gas. The obtained fragments were directed to the ICR mass analyzer and registered as a MS/MS spectrum. The collision voltage was optimized for the best fragmentation. For MS spectra analysis, a Bruker Compass DataAnalysis 4.0 software was used. A sophisticated numerical annotation procedure (SNAP) algorithm was used for finding the monoisotopic peaks. The mass accuracy error for all obtained signals was in the range of 2 ppm.

## Electronic supplementary material

Below is the link to the electronic supplementary material.

Supplementary caption 1 with my own citation
