# Supplementary material for: New method of peptide cleavage based on Edman degradation
Source: Mol Divers. 2013 May 21;17(3):605–11. doi: 10.1007/s11030-013-9453-y (PMC3713267; doi:10.1007/s11030-013-9453-y)
Supplement: Supplementary file 1 — Supplementary caption 1 with my own citation [file 11030_2013_9453_MOESM1_ESM.docx]

**Supporting materials**

**
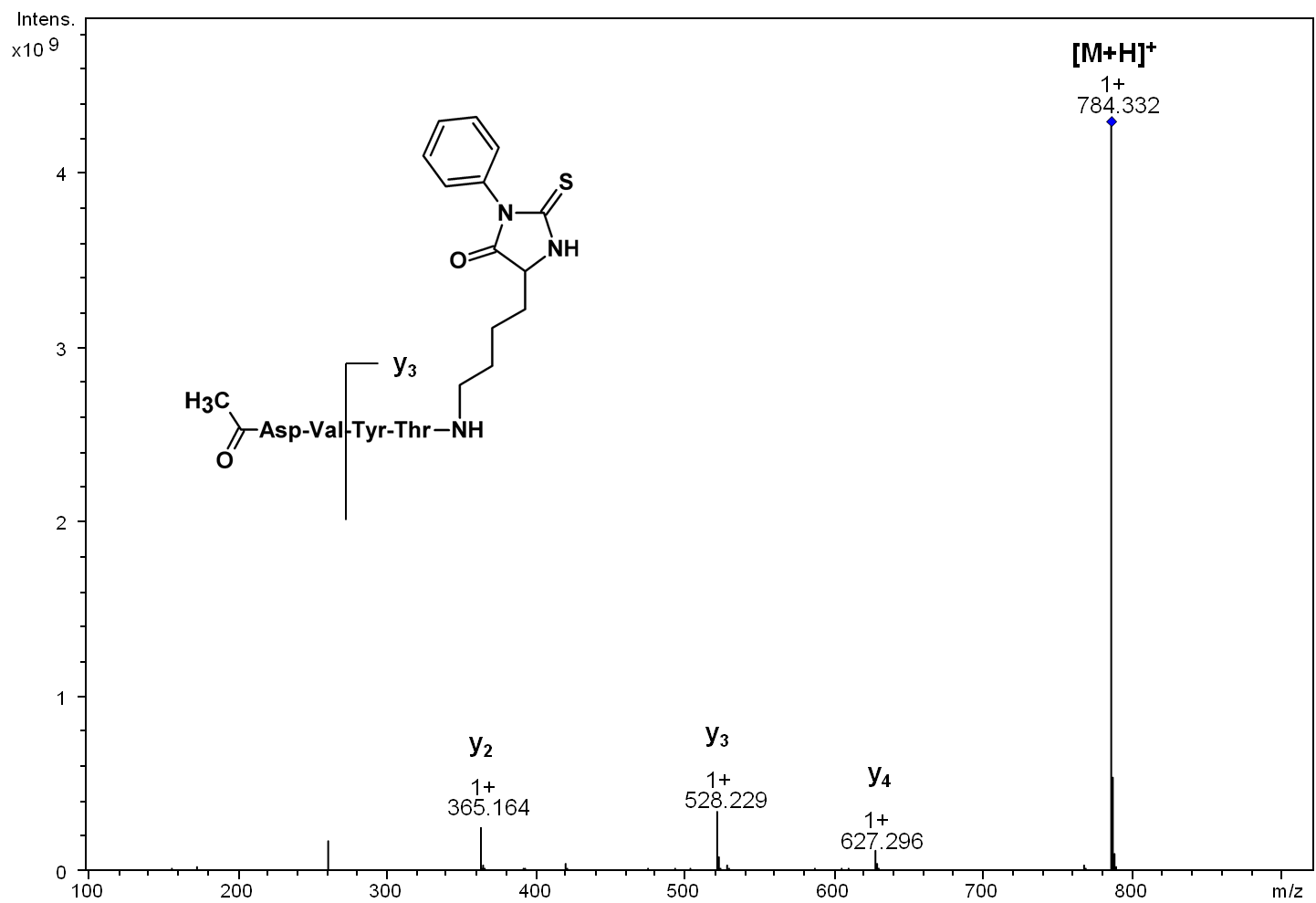
**

Spectrum S1. ESI-MS/MS spectrum of acetylated peptide cleaved from the TentaGel HL-NH_2_ resin in the form of PTH derivative.


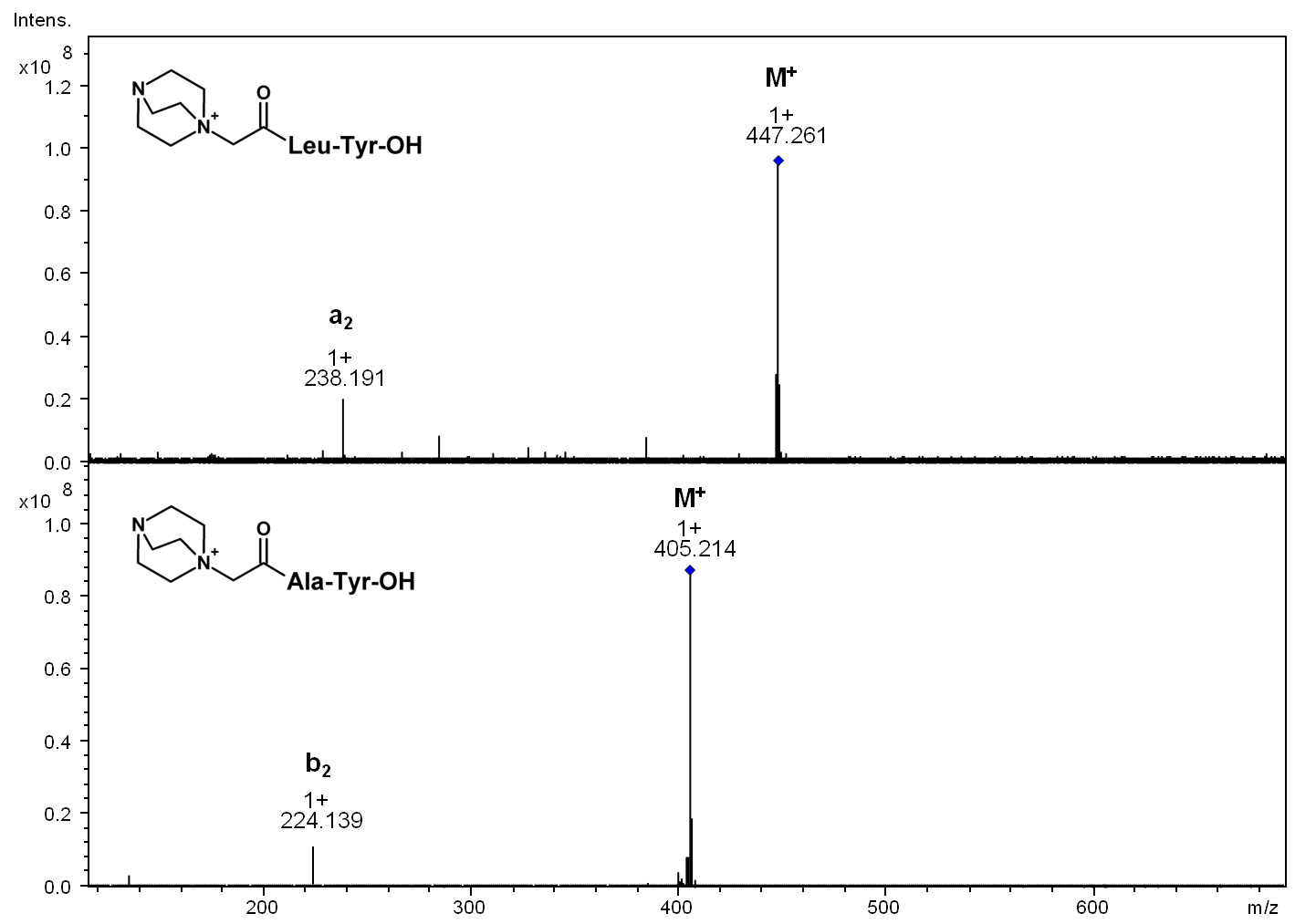


Spectrum S2. ESI-MS/MS spectra of fixed charge tagged peptides released by enzymatic digestion of single resin bead.

The ESI-MS/MS spectra of identified library components are presented below.


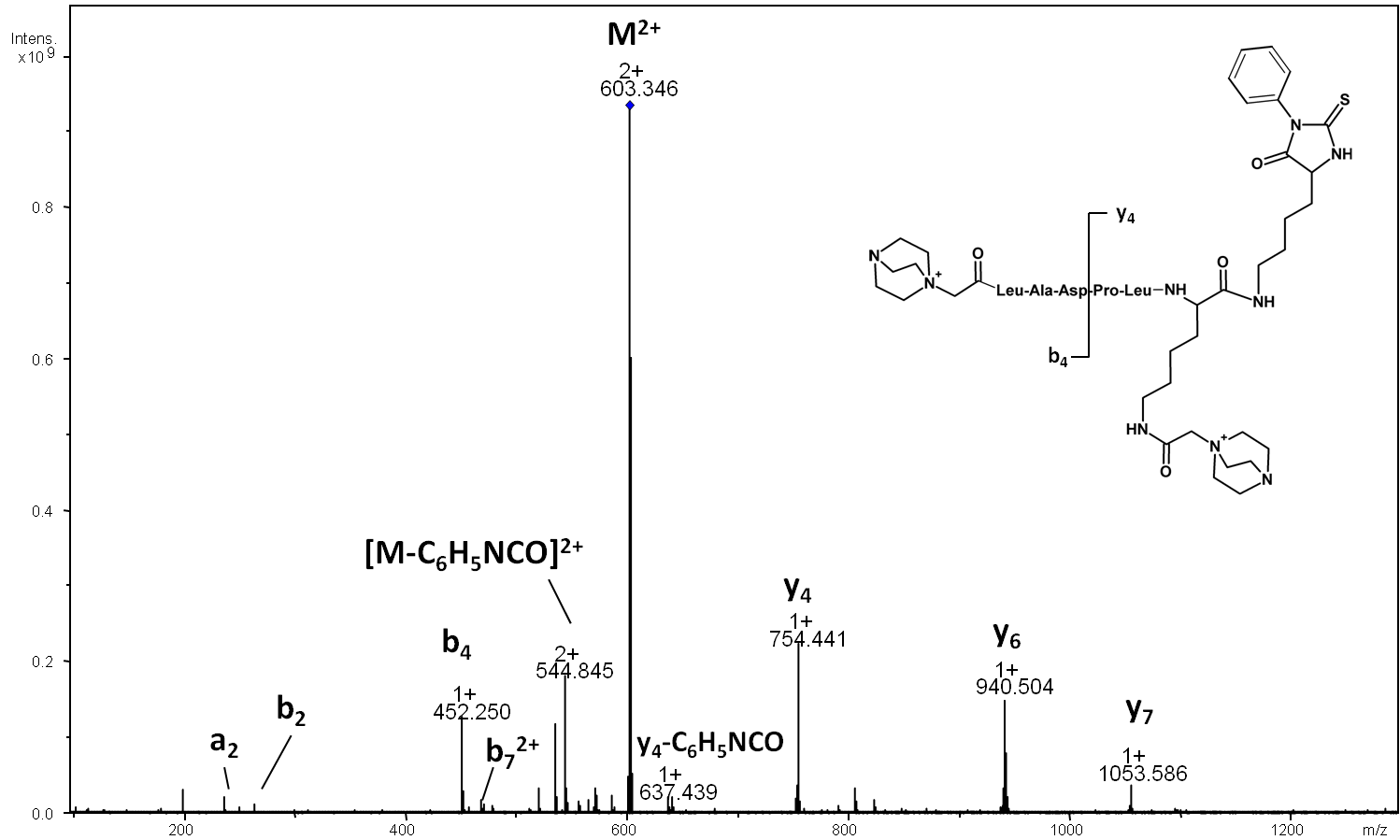


Spectrum S3. ESI-MS/MS spectrum of compound 1. The parent ion was 603.346 [M]^2+^.


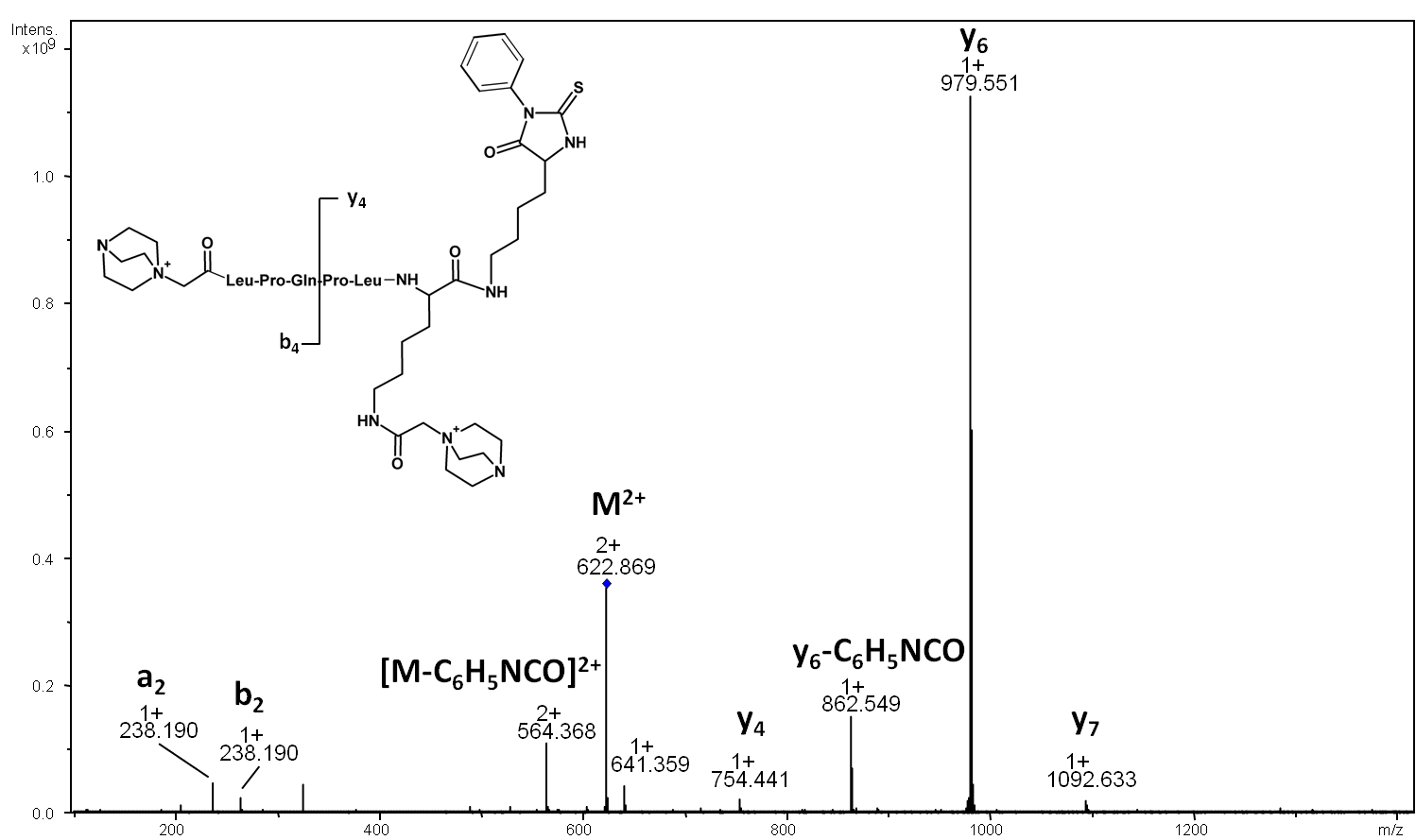


Spectrum S4. ESI-MS/MS spectrum of compound 2. The parent ion was 622.869 [M]^2+^.

**
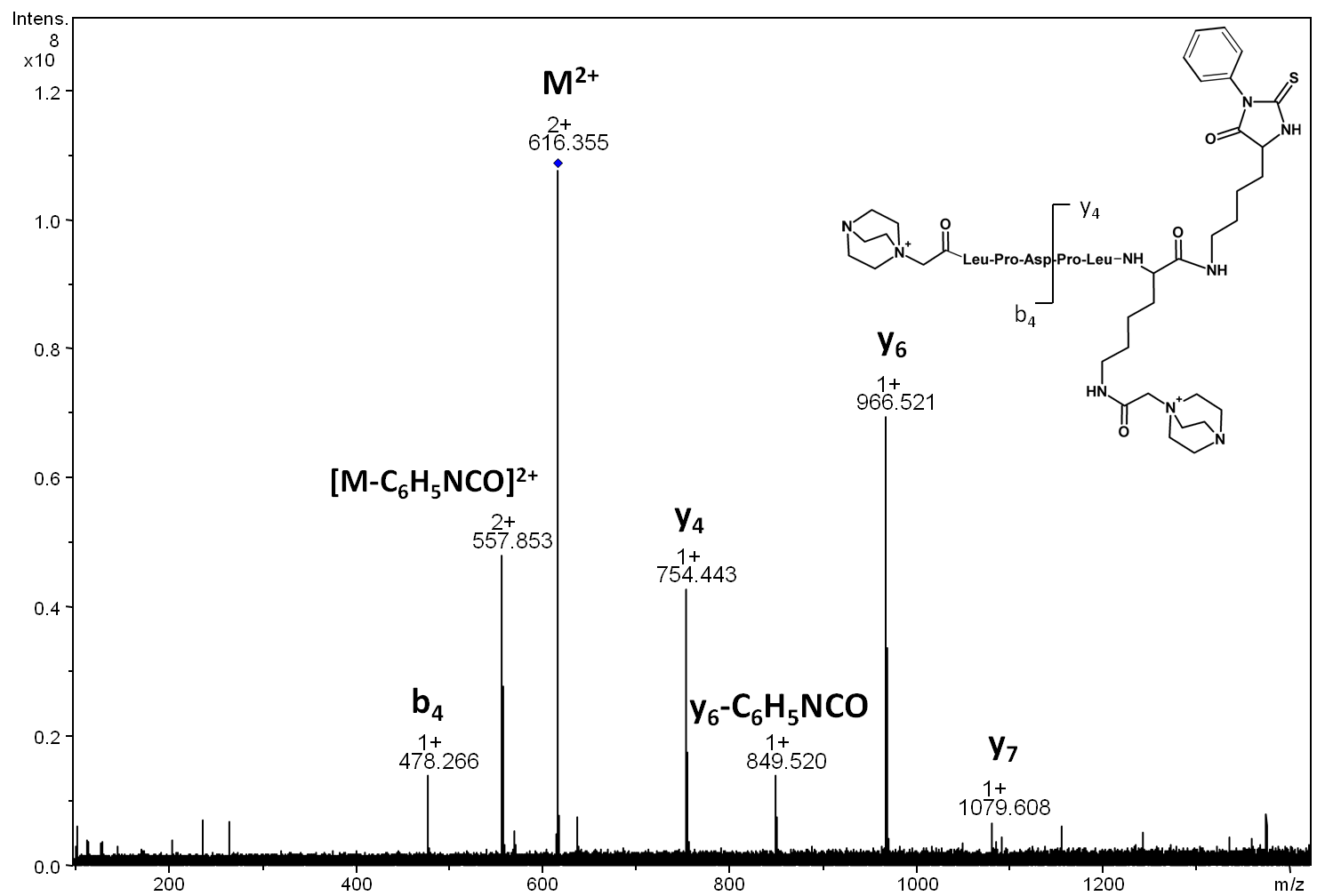
**

Spectrum S5. ESI-MS/MS spectrum of compound 3. The parent ion was 616.355 [M]^2+^.


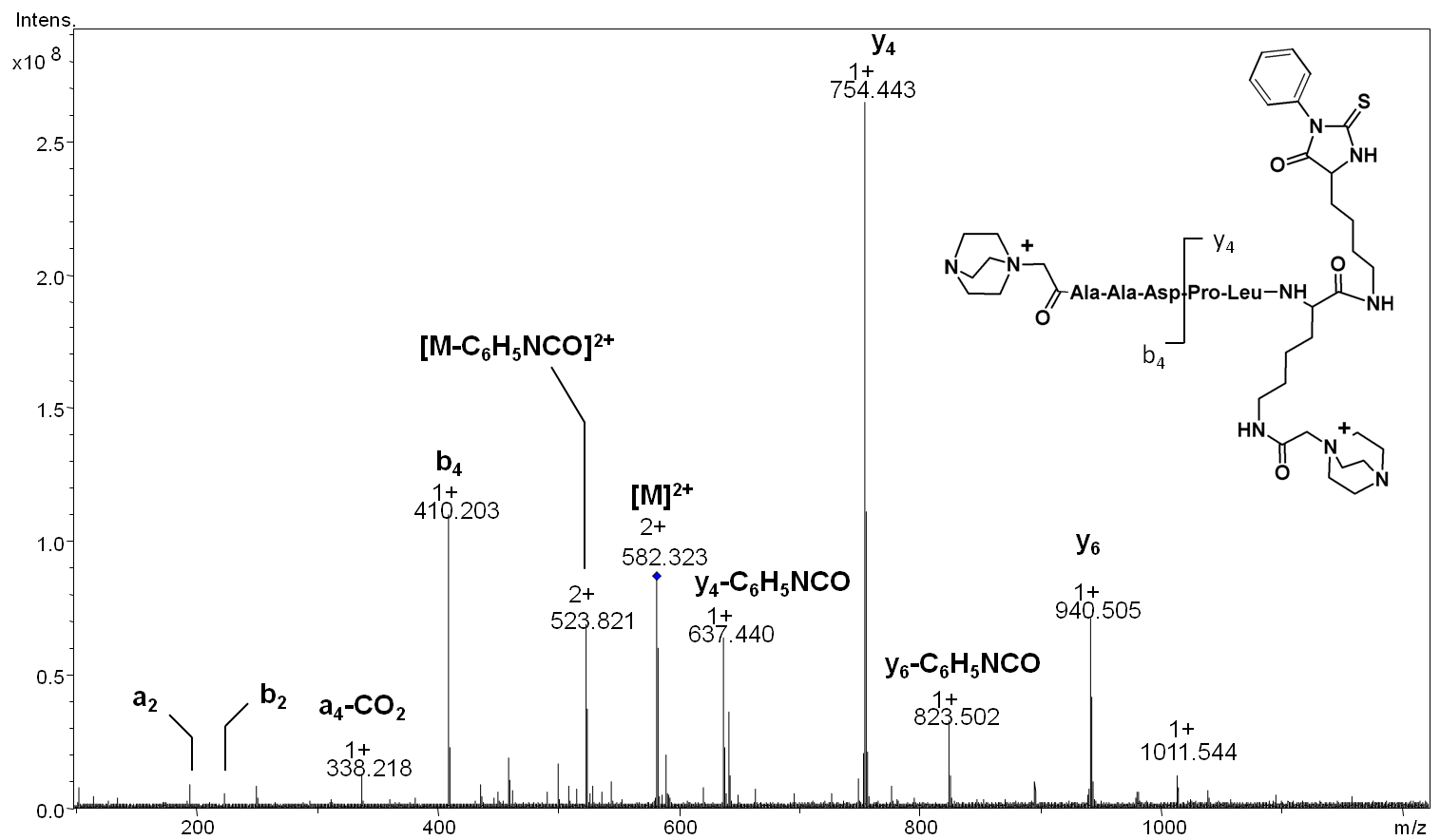


Spectrum S6. ESI-MS/MS spectrum of compound 4. The parent ion was 582.323 [M]^2+^.

**
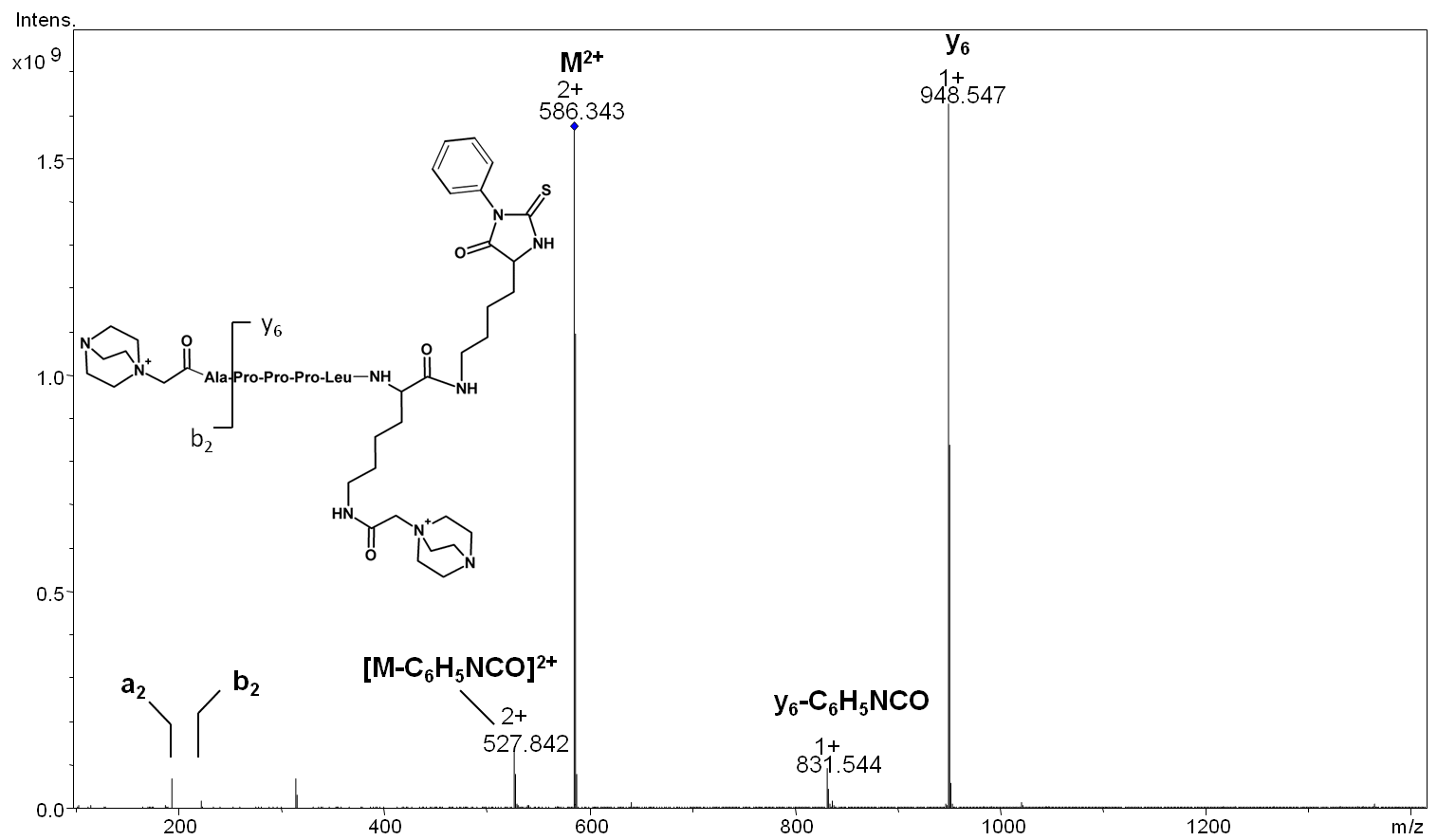
**

Spectrum S7. ESI-MS/MS spectrum of compound 5. The parent ion was 586.343 [M]^2+^.

**
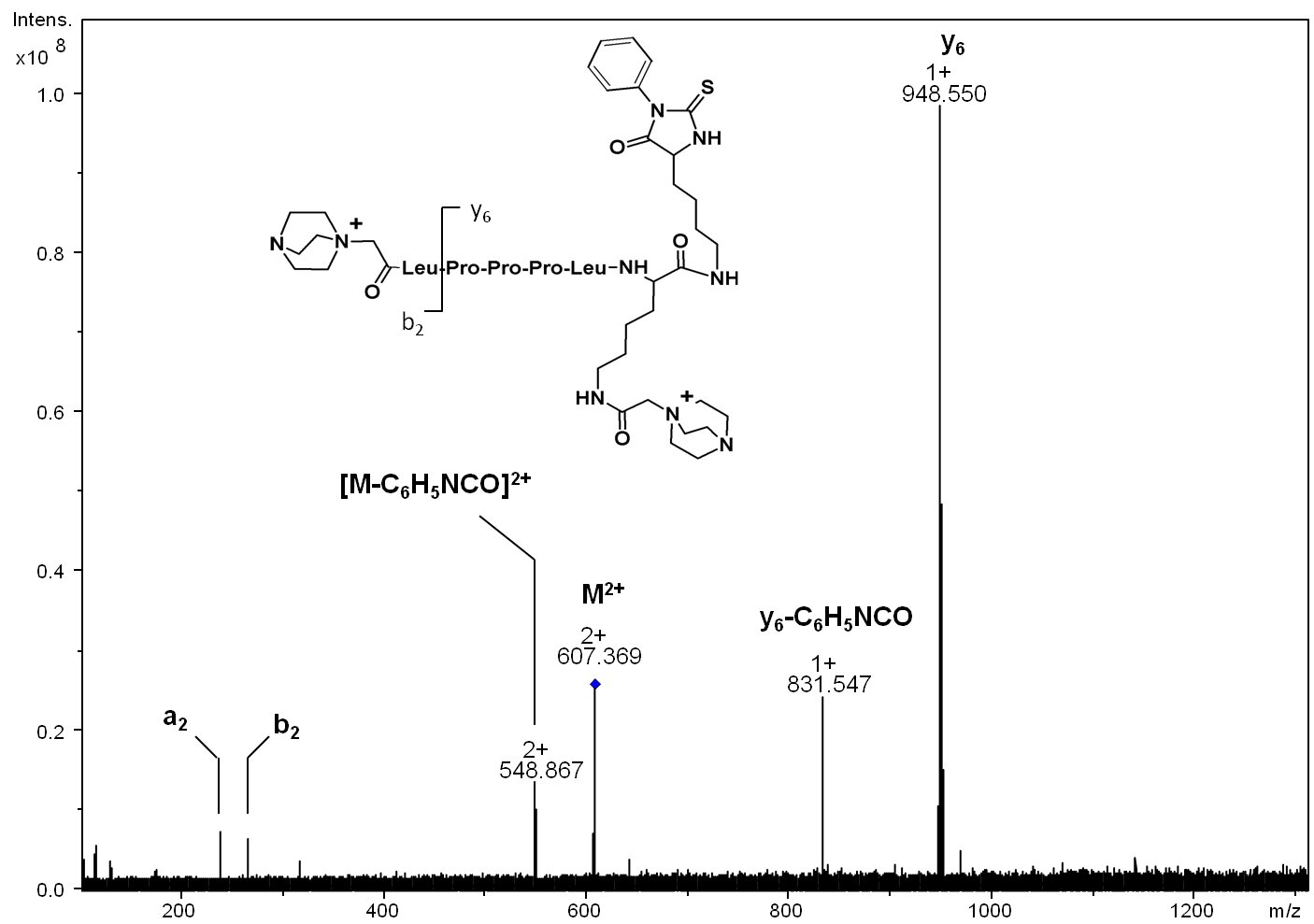
**

Spectrum S8. ESI-MS/MS spectrum of compound 6. The parent ion was 607.369 [M]^2+^.

**
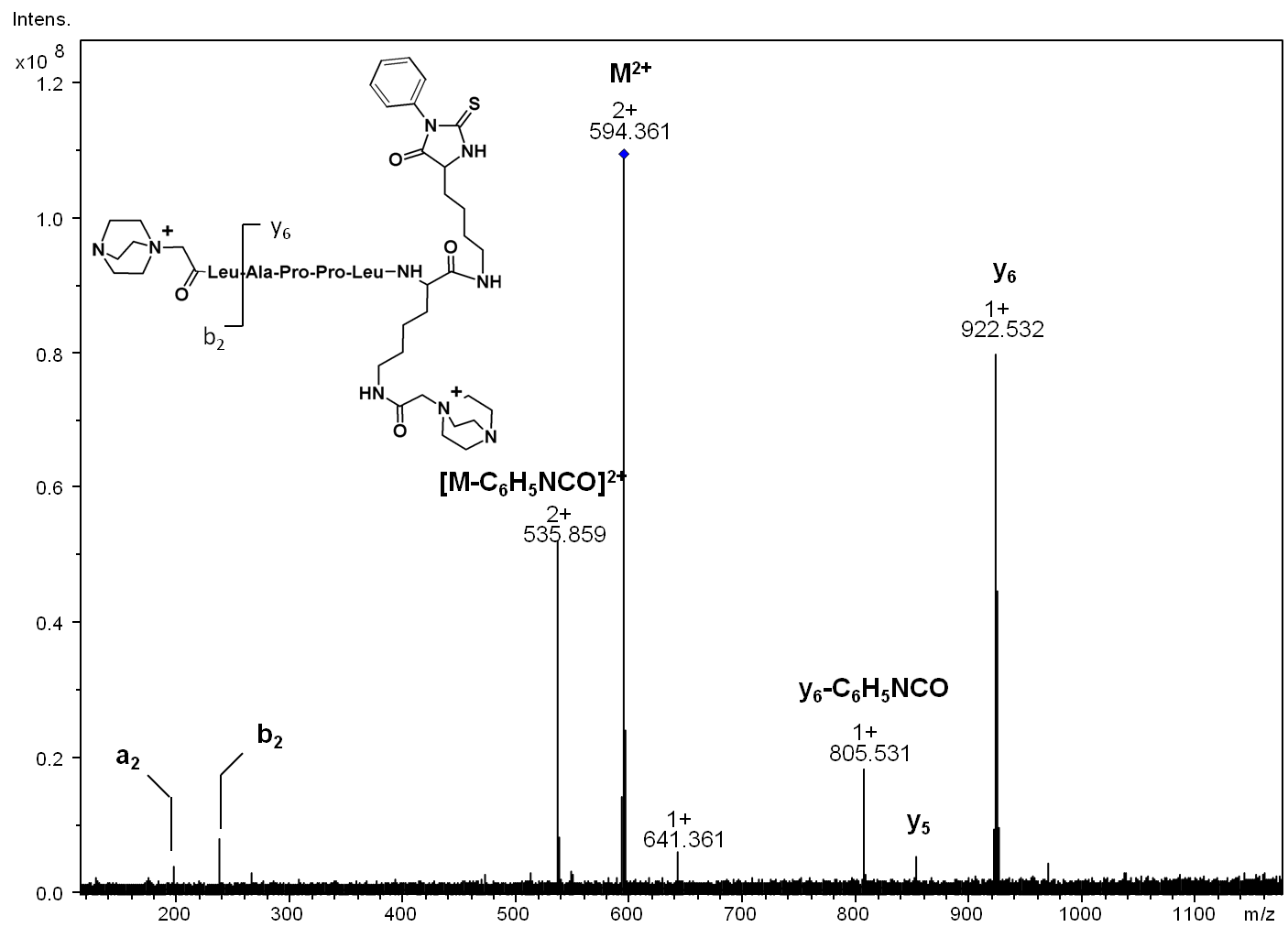
**

Spectrum S9. ESI-MS/MS spectrum of compound 7. The parent ion was 594.361 [M]^2+^.

**
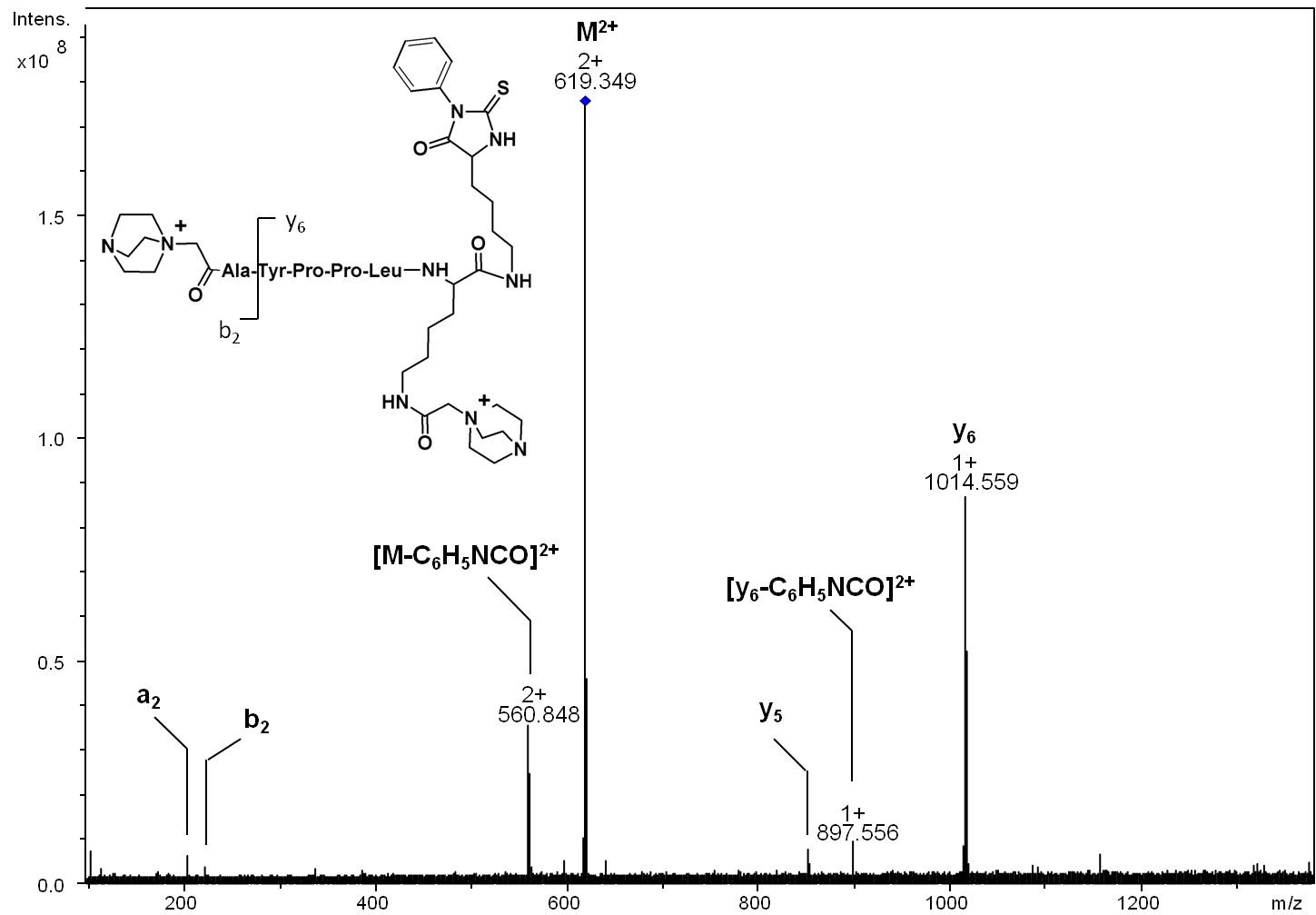
**

Spectrum S10. ESI-MS/MS spectrum of compound 8. The parent ion was 619.349 [M]^2+^.

**Peptide cleavage efficiency**

The results of elemental analysis presented in our report suggest that all the PTC modified peptides are removed from the resin, leaving no sulphur-containing residue. The TentaGel resin is designed to be resistant to standard cleavage procedures, therefore a special linker for CNBr cleavage has to be used to examine the completness of the cleavage by proposed method.


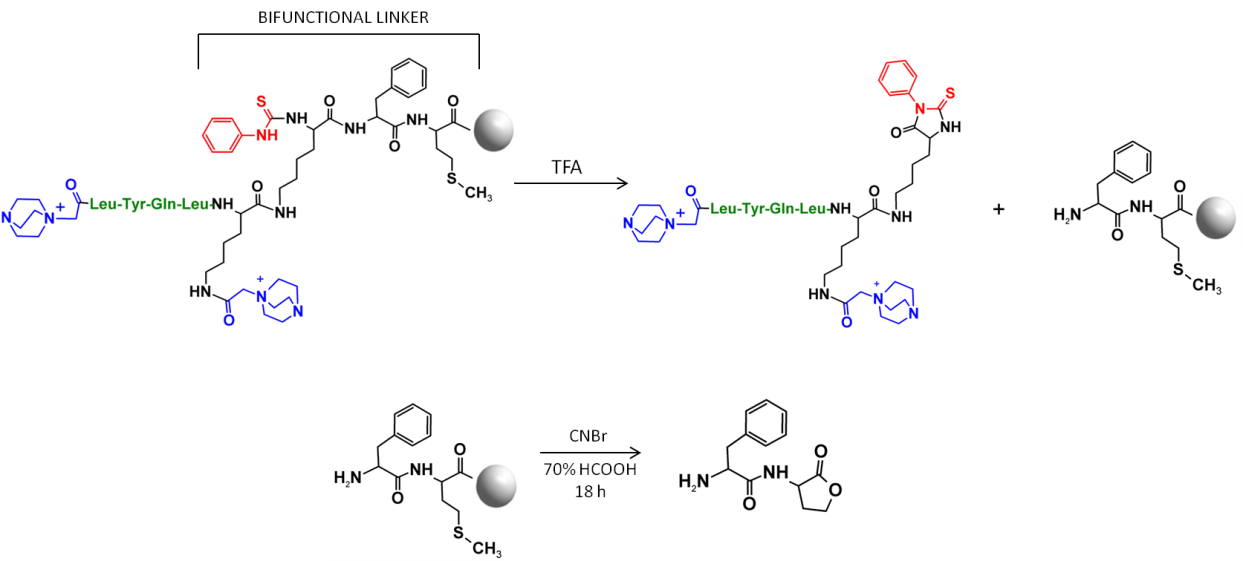


Figure S1. A new bifunctional linker used to analyze the cleavage efficiency. A grey ball represents a single resin bead.


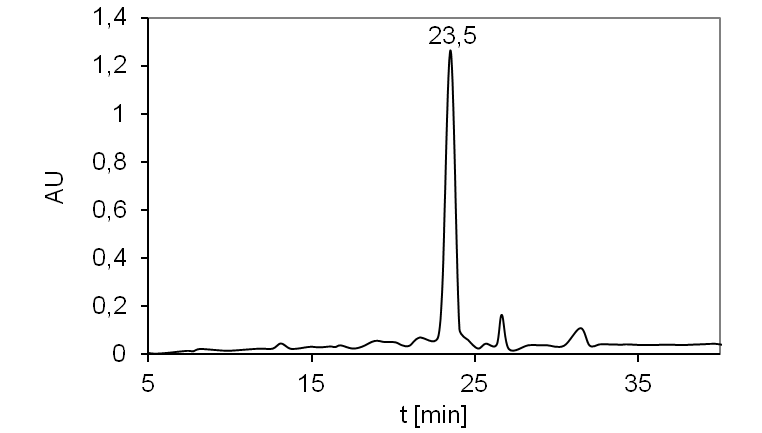


Figure S2. HPLC chromatogram of obtained product after 30’ incubation with TFA. The PTH-peptide product cleaved from the resin is characterized by RT=23.5 min.


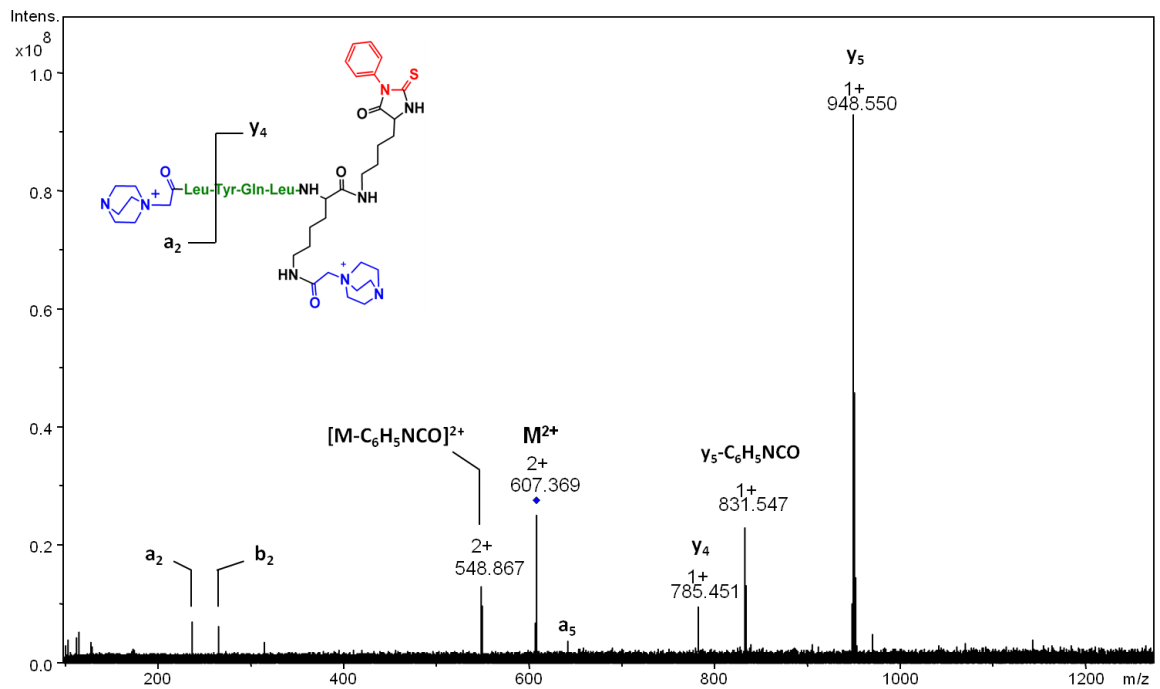


Spectrum S11. ESI-MS/MS spectrum of PTH-peptide derivative.

The remaining resin was then incubated with 0.25M cyanogen bromide in 70% HCOOH overnight to release the remaining peptide from the resin (Figure S3, Spectrum S11). Obtained results suggest that proposed cleavage method based on Edman degradation is highly efficient.


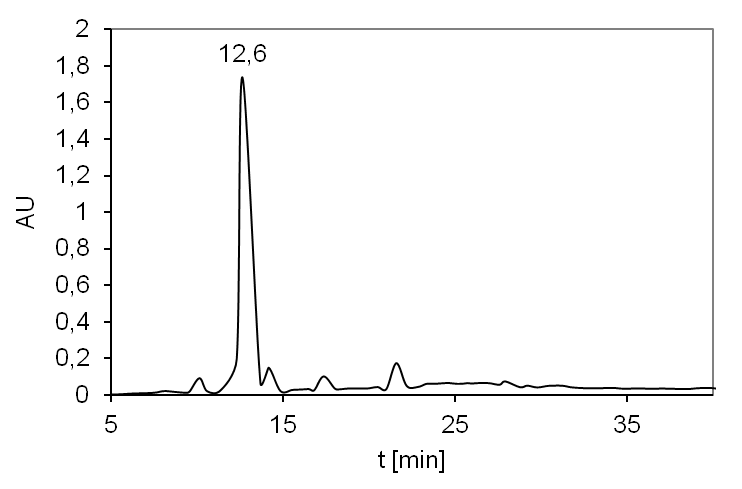


Figure S3. HPLC chromatogram of obtained product after incubation of remaining resin with 0.25M cyanogen bromide in 70% HCOOH overnight. The homoserine lactone derivative cleaved from the resin is characterized by RT=12.6 min.


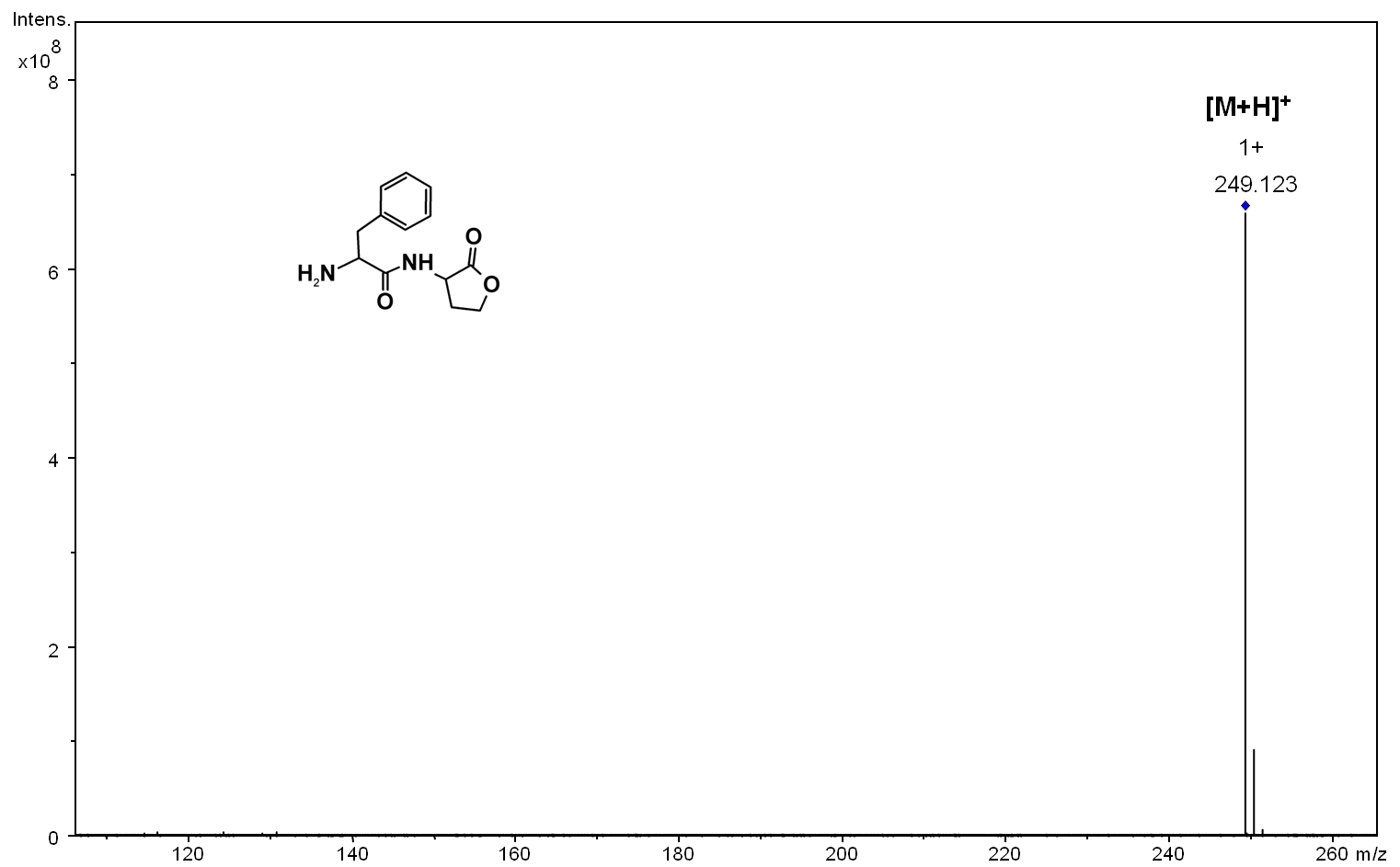


Spectrum S12. ESI-MS spectrum of homoserine lactone derivative.

The results of CNBr cleavage show that the reaction of PITC with α-amino group of lysine and subsequent cleavage by phenylthiohydantoin formation are quantitative.
